# The Deep Effects of the "X" Rays

**Published:** 1897-08-07

**Authors:** 


### The Deep Effects of the "X" Rays.

It is becoming increasingly evident that the Röntgen rays in traversing the tissues do something more than pass idly through. In our present ignorance as to the exact nature of this form of "radiation," we must refrain from any speculation as to what happens to the "rays" in their course; but it seems clear that in their passage they do exercise a selective and sometimes a more or less destructive influence on certain tissues.

Dr. David Walsh has recently published some very interesting observations on this point. Many cases have, of course, been described in which a more or less severe dermatitis, sometimes leading to obstinate ulceration, has followed the use of the rays, and an American surgeon has recorded a case in which this was accompanied by an affection of the bones of the hand which had been exposed. In one of Dr. Walsh's cases, after a prolonged demonstration, in which the tube had been constantly near the head, although separated by a wooden screen, the patient suffered from giddiness, headache, vomiting, diarrhœa, high temperature, and prostration. In another the patient was carrying out a series of experiments involving the exposure of the region of the stomach for about two hours daily. After some weeks gastric symptoms occurred, which were removed by change of air and cessation of work. They soon returned, however, on his resuming his experiments, but ceased on his shielding the affected region with a thin sheet of lead.

In a case related by Professor Reid, of Dundee, in which severe dermatitis and loss of hair followed exposure of the chest and belly to the rays, redness of the back also occurred, showing that the rays after passing through the body still possessed a kind of selective and irritative action. Whether such action can be utilised as a therapeutic agent is a deeply interesting question. Several experiments have been made with the object of ascertaining whether the rays could be made to influence tuberculosis, but the recent reports to the Académie de Médecine in Paris are not such as to give much encouragement to the idea.

The matter, however, is full of interest in other directions besides crude attempts to kill bacilli. The relation of the "X" or other analogous "rays" to sunlight is a matter of extreme importance. It has, perhaps, been too hastily assumed that clothed mankind is indifferent to sunlight, and that where he has seemed to benefit by a "sun bath" the benefit has been the result of the bactericidal action of the actinic rays, or the production of ozone or some other more or less hypothetical attribute of sunshine. It must be remembered that we only know of the "X" rays because their chemical action on certain substances and their fluorescent effect on others have been discovered. We have, however, but little reason to believe that the "X" rays are one and indivisible; and, in fact, the difficulty of inducing the same tube to give always the same results, points strongly to the fact that they are many and various, and that little as we know about the "X" rays, there is a whole alphabet of other so-called rays about which we know absolutely nothing.

Who is to say, then, that along with the manifest forms of energy, heat, light, and actinism which we know to beat upon this earth in the form of sunshine, other forms of energy do not exist of whose presence we so far see no sign; perhaps riding along with the undulations which we know of, perhaps only developed at the moment of impact, when the undulations become manifest by their influence on material substances, but in either case possessing powers of penetration and activity analagous with those "rays" or "radiations" which, for want of more complete knowledge, we still designate by the symbol "X." These are matters in regard to which ignorance at present prevails. *Quien sabe!* Sometime perhaps we shall understand why plums ripen through to the middle, and why the sunshine does us good.

